# Room-tem­per­a­ture crystal structures of [CH(NH_2_)_2_]_3_Sb_2_*X*_9_ (*X* = Br and I)

**DOI:** 10.1107/S2053229626000811

**Published:** 2026-02-26

**Authors:** Prajna Bhatt, Yuhan Liu, Anna Regoutz, Robert G. Palgrave

**Affiliations:** a Department of Chemistry, University College London, 20 Gordon St, London, WC1H 0AJ, United Kingdom; b Istituto Officina dei Materiali (IOM)–CNR Area Science Park SS14 Km 1635 Trieste I-34149 Italy; c The Electrochemical Innovation Lab, Department of Chemical Engineering, University College London, Torrington Place, London, WC1E 7JE, United Kingdom; d Department of Chemistry, University of Oxford, Inorganic Chemistry Laboratory, South Parks Road, Oxford, OX1 3QR, United Kingdom; University of North Texas at Dallas, USA

**Keywords:** halide per­ov­skite, crystallography, counter diffusion crystal growth, CDCG, single crystal, crystal structure, formamidinium, anti­mony

## Abstract

Crystals of formamidinium anti­mony, halides, FA_3_Sb_2_*X*_9_ {FA = [CH(NH_2_)_2_]^+^; *X* = Br^−^ and I^−^} {or triformamidinium nona­halido­dianti­mony, (CH_5_N_2_)_3_[Sb_2_*X*_9_]}, have been synthesized using a counter diffusion crystal growth (CDCG) method in silica gel and their structures determined from single-crystal X-ray diffraction data. FA_3_Sb_2_Br_9_ belongs to the trigonal space group *P*3*m*1, which is known as the Cs_3_Bi_2_Br_9_ structure type, and FA_3_Sb_2_I_9_ belongs to the hexa­gonal space group *P*6_3_/*mmc*, called the Cs_3_Cr_2_Cl_9_ structure type. The change of the anion type from bromide to iodide results in the change of the structure type and the connectivity of the Sb—*X* octa­hedra. These structures are described and com­pared to the crystal types known for vacancy-ordered triple-per­ov­skites.

## Abstract 2

Two new vacancy-ordered formamidinium anti­mony halide triple-per­ov­skites, FA_3_Sb_2_*X*_9_ {FA = [CH(NH_2_)_2_]^+^; *X* = Br and I}, measured by single-crystal X-ray diffraction are described.

## Introduction

The *A*_3_*B*_2_*X*_9_ structure, where *A* is a monovalent cation, *B* is a trivalent cation and *X* is a halide, are commonly described as derivatives of the *ABX*_3_ per­ov­skite structure type. In the *ABX*_3_ form, all [*BX*_6_]^3−^ octa­hedra are corner-sharing and an *A*_3_*B*_2_*X*_9_ com­pound can be considered similarly, with removal of a third of the *B*-site cations (Hodgkins *et al.*, 2019 ▸; Chang *et al.*, 2016 ▸). As such, these structures are more commonly known as vacancy-ordered triple-per­ov­skites. Typical *A*_3_*B*_2_*X*_9_ com­pounds are group 15 halides, mostly com­prising of bis­muth (Bi) and anti­mony (Sb) *B*-sites. The triple-per­ov­skites are considered as per­ov­skite derivatives that can similarly be applied in photovoltaic and radiation detection applications as lead-free alternatives (Eperon *et al.*, 2014 ▸; Hao *et al.*, 2014 ▸). Due to the optical and electronic properties possessed by these com­pounds for such applications, materials design approaches also include the formation of com­pounds with organic monovalent cations. Here, the crystal structure of two organic–inorganic triple-per­ov­skites, namely, FA_3_Sb_2_*X*_9_ {FA = [CH(NH_2_)_2_]^+^; *X* = Br^−^ and I^−^}, are reported.

## Experimental methods

Single crystals were synthesized using a counter diffusion crystal growth (CDCG) method in silica gel. 27 mmol of Sb_2_O_3_ (Sigma–Aldrich, 99%) were reacted with excess H*X*, where *X* = Br (Sigma–Aldrich, 48 wt%) or I (Sigma–Aldrich, 57 wt%), to produce Sb*X*_3_ in an acidic solution. In parallel, a 0.6 *M* aqueous solution of Na_2_SiO_3_ (Sigma–Aldrich) was prepared using distilled water. The Na_2_SiO_3_ solution was added dropwise in the presence of vigorous stirring to Sb*X*_3_ in a 1:1 (*v*/*v*) ratio to form a Sb*X*_3_-based silica gel. The solution was allowed to set in 50 ml tall-form beakers in a low-tem­per­a­ture oven at 29 °C over 24 h. Post gelation, solutions containing 41 mmol of FA*X* {FA = [CH(NH_2_)_2_]^+^}, made by dissolving formamidine acetate (Sigma–Aldrich, ≥ 98%) in H*X*, were added carefully atop the gel using pipettes to avoid disrupting the surface of the gel. The beaker was wrapped with parafilm and placed in an oven. Crystal growth occurred between 2–7 d. FA_3_Sb_2_Br_9_ was isolated in the form of pale-yellow plate-like crystals, while FA_3_Sb_2_I_9_ crystallized in the morphology of red–brown needle-like crystals (Fig. S1).

Powder X-ray diffraction (PXRD) was measured with a Stoe STADI-P X-ray diffractometer in thin foil transmission (Debye–Scherrer geometry) mode equipped with a germanium (111) monochromator and a Dectris Mythen 1K detector, with Cu *K*α (λ = 1.5406 Å, using 40 kV and 30 mA) radiation at 298 K. Samples were loaded between two clear acetate sheets and sealed using silicon vacuum grease. Diffraction patterns were collected in a 2θ range from 2 to 70°, with a step size of 0.015° and a scan rate of 5 s per step. Rietveld refinement models (Rietveld, 1966 ▸) on PXRD data were carried out within the *TOPAS-Academic* software suite (Version 7; Coelho, 2018 ▸; Coelho, 2022 ▸).

Single-crystal X-ray diffraction (SCXRD) was performed using an Agilent SuperNova diffractometer with an Atlas CCD detector. Full spheres of data were collected using 1° scan frames in ω with monochromated Cu or Mo *K*α radiation at 295 K. A refinement of the positions of the C, N and H atoms was not carried out because isotropic rotation of the FA cations takes place at room tem­per­a­ture like other organic monovalent cations (Liu *et al.*, 2022 ▸). The supporting information includes a discussion on the treatment of the FA cation in detail. The experimental details from SCXRD are sum­mar­ized in Table 1 ▸.

All other experimental techniques employed (Raman spectroscopy, X-ray photoelectron spectroscopy and diffuse reflectance spectroscopy) are described in the supporting information.

## Results and discussion

### Description of structures

#### Formamidinium anti­mony bromide, FA_3_Sb_2_Br_9_

FA_3_Sb_2_Br_9_ was found to crystallize in the Cs_3_Bi_2_Br_9_ structure type. Compounds of this crystal type belong to the trigonal space group *P*
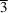
*m*1 (Laza­rini, 1977 ▸). The lattice parameters for FA_3_Sb_2_Br_9_ are *a* = 8.5161 (4), *c* = 10.0380 (4) Å and *V* = 630.46 (6) Å^3^. Similar to most organic–inorganic triple-per­ov­skites, the FA cations possess rotational disorder at room tem­per­a­ture (Bhatt *et al.*, 2025 ▸), resulting in failed attempts to localize discrete C and N atoms. The solvent-masking routine in *OLEX2* was employed (Dolomanov *et al.*, 2009 ▸) and a void volume of 244 Å^3^ per unit cell identified (38.7% of the total unit cell). The integrated electron count within this void was found to be 77 electrons. This is in excellent agreement with the 75 electrons expected theoretically for the three FA cations in the unit cell, confirming the existence of FA cations in the com­pound. In this article, a single C atom was used as a placeholder in the unit cell to represent the FA cation, as discussed in the methods and supporting information.

**Figure 1:**
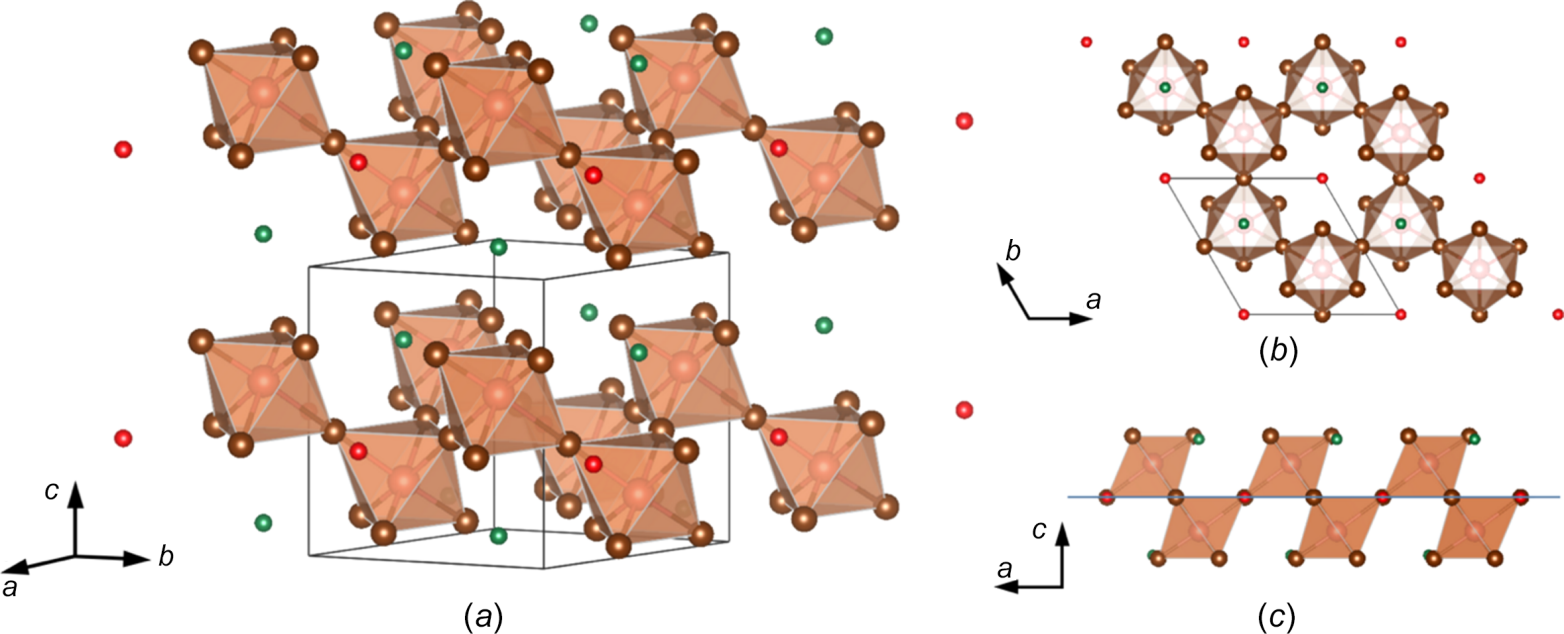
Representation of FA_3_Sb_2_Br_9_ from this work shown (*a*) obliquely, (*b*) down the *c* axis and (*c*) down the *b* axis. Four unit cells are shown, with a black-bordered box around one unit cell. Pink spheres represent Sb, brown spheres represent Br and the light-brown octa­hedra represent the [SbBr_6_]^3−^ coordination environment. *A*-site atoms are bifurcated by their Wyckoff positions, *i.e.* green spheres represent C atoms on site 2*d* between [SbBr_6_]^3−^ layers and red spheres reside on site 1*a* between rings of six [SbBr_6_]^3−^ octa­hedra. C atoms are used in place of the FA ion, as explained in the text. The blue layer in (*c*) represents the common plane of bromide ions, as explained in the text. The figure was prepared using the *VESTA* software suite (Version 3; Momma & Izumi, 2011 ▸).

Common bromide-based vacancy-ordered triple-per­ov­skites that are also known to crystallize in this structure type include MA_3_Bi_2_Br_9_, MA_3_Sb_2_Br_9_ and FA_3_Bi_2_Br_9_ (MA = CH_3_NH_3_^+^) (Ishihara *et al.*, 1992 ▸; Tomaszewski, 1994 ▸; Shen *et al.*, 2020 ▸). The isostructural Cs_3_Bi_2_Br_9_ was reported to have the Cs and Br atoms in a cubic close-packed arrangement, with the Bi atom occupying one-sixth of the octa­hedral holes in the crystal structure. The structure of FA_3_Sb_2_Br_9_ can also be considered a ‘2D form’ of a vacancy-ordered triple-per­ov­skite (Chen *et al.*, 2024 ▸). Here, the corner-sharing [SbBr_6_]^3−^ octa­hedra possess a structural dimensionality such that the octa­hedra can be described as 2D layers, within which *A*-site cations are located. For FA_3_Sb_2_Br_9_ visualized in Fig. 1 ▸(*a*), [SbBr_6_]^3−^ octa­hedra (in light brown) generate these 2D layers by sharing three Br atoms with three different neighbouring octa­hedra.

Within the layer, the corner-sharing octa­hedra result in the formation of six-connected octa­hedra ‘rings’ [Fig. 1 ▸(*b*)] where the FA ions reside (red atoms). This is similar to the *ABX*_3_ per­ov­skite structure with the exception of six surrounding *B*-site octa­hedra rather than eight (Xia *et al.*, 2020 ▸). In addition, the FA ions fill spaces between these corner-sharing octa­hedra 2D layers (green), observed as [SbBr_6_]^3−^–FA^+^–[SbBr_6_]^3−^ layers along the *c* axis. The *A*- and *B*-site coordination numbers are 12 and 6, respectively, and agree with other com­pounds that crystallize in this structure type.

This structure has also been described previously as isolated layers formed by [SbBr_6_]^3−^ octa­hedra pointing alternatively up or down with respect to a plane of common halide atoms (Tomaszewski, 1994 ▸). Fig. 1 ▸(*c*) shows the orientation of the octa­hedra alternating in orientation with respect to the layer of bromide ions (displayed with a blue line).

Refined atomic position parameters, selected inter­atomic distances and bond angles for FA_3_Sb_2_Br_9_ are listed in Table 2 ▸. Each [SbBr_6_]^3−^ octa­hedron has three equivalent Sb—Br1 and Sb—Br2 distances of 3.0598 (7) and 2.612 (2) Å, respectively. These are com­parable to the distances reported in MA_3_Sb_2_Br_9_, where Sb—Br1 = 3.000 Å and Sb—Br2 = 2.627 Å (Ishihara *et al.*, 1992 ▸). Both FA_3_Sb_2_Br_9_ and MA_3_Sb_2_Br_9_ have similar bond angles of Br1—Sb—Br2 (≈177°) and Br*x*—Sb—Br*x* (in the range 88–92°), where *x* is the same number. This difference in the Sb—Br bond lengths and angles within a single octa­hedron arises from the distortions from the vertex-sharing Br1 halide ions.

When com­pared to the bis­muth com­pound MA_3_Bi_2_Br_9_, the Sb—Br1 distance of FA_3_Sb_2_Br_9_ is similar to Bi—Br1 (3.054 Å), while Sb—Br2 of FA_3_Sb_2_Br_9_ is smaller than Bi—Br2 (2.772 Å) in MA_3_Bi_2_Br_9_. The six-coordinate ionic radius of Sb^3+^ (0.76 Å) in FA_3_Sb_2_Br_9_ is smaller com­pared to that of Bi^3+^ (1.03 Å) in MA_3_Bi_2_Br_9_ (Ahrens, 1952 ▸; Shannon, 1976 ▸). Anti­mony com­pounds are therefore expected to have smaller unit cells and shorter bond lengths com­pared to bro­mo­bis­muthates. Smaller *B*-site ions also mean the [SbBr_6_]^3−^ octa­hedra are expected to have lower angular distortions than [BiBr_6_]^3−^. Considering bond angles, there is a stronger distortion of the Br—Bi—Br angles, with Br1—Bi—Br2 being ≈169.3° and Br*x*—Bi—Br*x* in the range 84–88°. The angles in the Bi com­pound are smaller than equivalent octa­hedral bond angles in FA_3_Sb_2_Br_9_, where Br atoms are arranged around a larger cation (Laza­rini, 1977 ▸).

**Figure 2:**
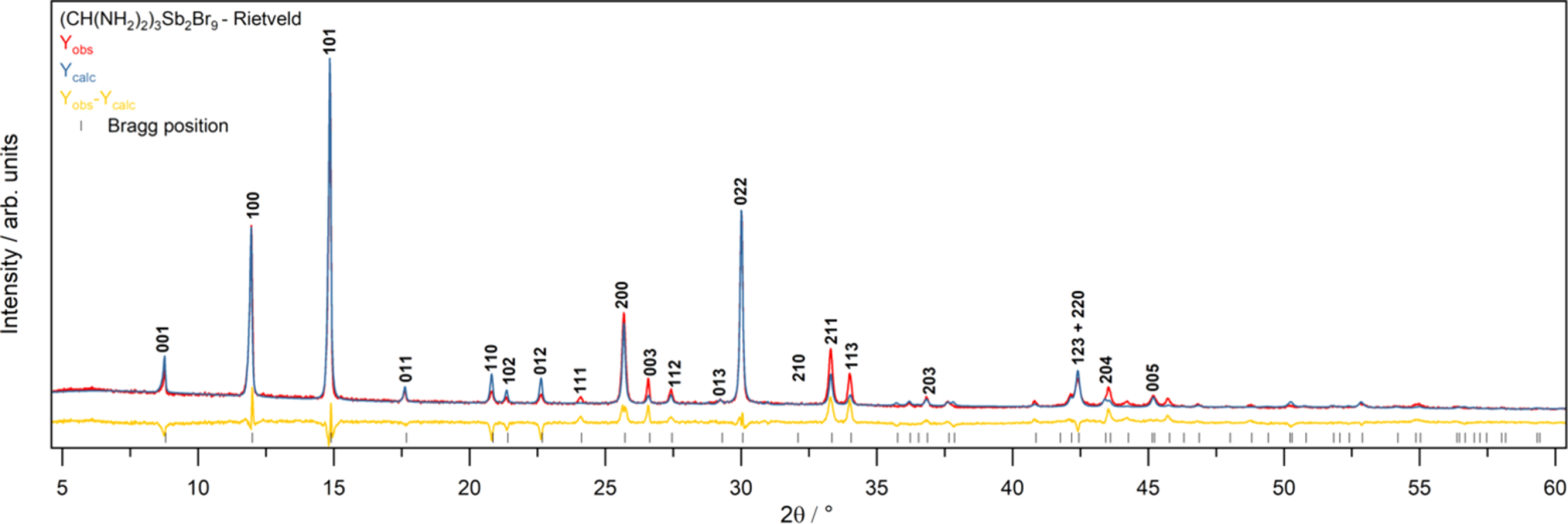
Rietveld refinement on the PXRD pattern of FA_3_Sb_2_Br_9_ single crystals made by CDCG. Input unit-cell information was taken from the structure resolved from SCXRD data. Y_obs_ (red) is the collected diffraction pattern, Y_calc_ (blue) is the calculated pattern from *TOPAS-Academic* and Y_obs_ − Y_calc_ (yellow) is the residual plot. Reflections that are ≥5% of the highest intensity reflection are indexed.

The plotted Rietveld refinement of PXRD data (Fig. 2 ▸) shows excellent agreement between the Y_obs_ and Y_calc_ plots. The commensurate position of the Bragg reflections from SCXRD resolution and the peak positions from PXRD data indicate that the structural resolution is accurate for both single crystals and polycrystalline powders. A goodness-of-fit (GOF) of 1.14 and *R*_w_ = 10.319% were achieved.

#### Formamidinium anti­mony iodide, FA_3_Sb_2_I_9_

**Figure 3:**
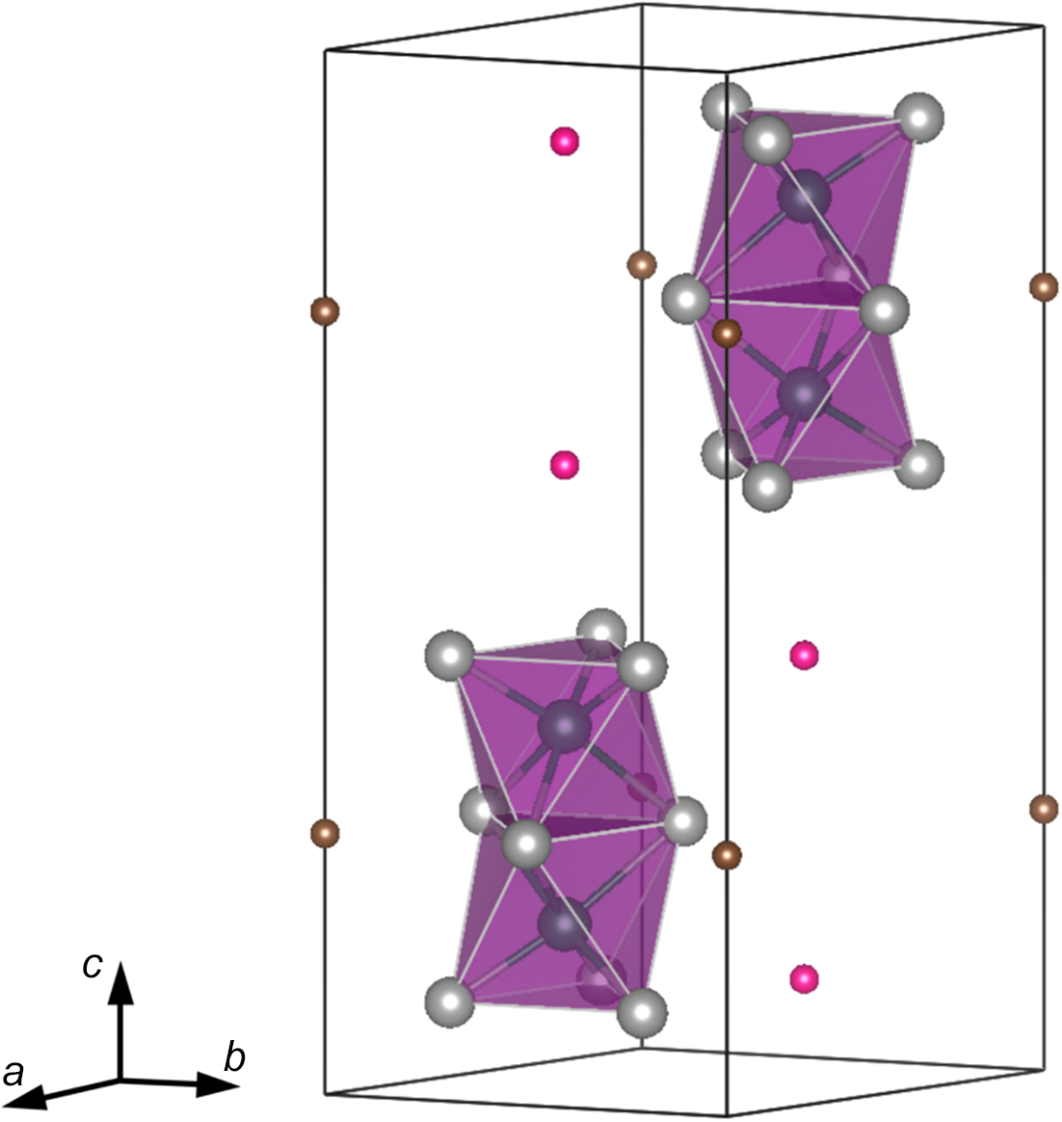
Unit-cell representation of FA_3_Sb_2_I_9_ from this work shown obliquely. Green spheres represent Sb, grey spheres represent I and purple bi­octa­hedra represent the [Sb_2_I_9_]^3−^ coordination environment. C atoms are bifurcated by their Wyckoff positions, *i.e.* pink spheres represent C atoms on site 4*f* and brown spheres reside on site 2*b*. C atoms are used in place of the FA ion, as explained in the text. The figure was prepared using the *VESTA* software suite (Version 3; Momma & Izumi, 2011 ▸).

FA_3_Sb_2_I_9_ belongs to the hexa­gonal space group *P*6_3_/*mmc* at room tem­per­a­ture. This is known as the Cs_3_Cr_2_Cl_9_ structural type wherein FA_3_Bi_2_I_9_, MA_3_Sb_2_I_9_ (MA = CH_3_NH_3_), Cs_3_Sb_2_I_9_ and Cs_3_Bi_2_I_9_ are known structures of group 15 triple-per­ov­skites (Szklarz *et al.*, 2019 ▸; Ju *et al.*, 2018 ▸; Yamada *et al.*, 1997 ▸; Arakcheeva *et al.*, 1999 ▸). FA_3_Sb_2_I_9_ has been reported previously at 195 K, also exhibiting the space group *P*6_3_/*mmc* (Szklarz *et al.*, 2020 ▸). One way to describe this structure considers the FA cation and I atoms forming close-packed FAI_3_ layers, with Sb atoms occupying one-sixth of the total octa­hedral holes (Arakcheeva *et al.*, 1999 ▸). The close packing of the *AX* layers is hexa­gonal and the layered structure is com­prised of isolated [Sb_2_I_9_]^3−^ bi­octa­hedra or ‘dimers’ which share a triangular face and three iodide ions. These are illustrated in Fig. 3 ▸ as purple polyhedra. As such, this variant of the triple-per­ov­skite is also regarded as a 0D isolated dimer structure. The Sb—Sb axis within each bi­octa­hedron is parallel to the *c* axis.

As in the bromide structure, the FA cations were found to be highly disordered. A total void volume of 541 Å^3^ (37.3% of the total unit cell) was identified, containing 105 electrons per unit cell, which corresponds to the region occupied by the disordered cations. While this is lower than the ideal count for six FA cations (150 electrons), it is consistent with the presence of rotationally disordered organic species that cannot be resolved into discrete atomic positions. When a single C atom in the void replaces one FA cation, a similar *R* factor is achieved. Thus, the FA cations in the unit cell are repre­sent­ed by C atoms.

The C1 position of the cation (pink) at Wyckoff position 4*f* is in the same plane of each dimer and its coordination can be described as identical layered stacking of the iodide ions with a hexa­gonal coordination (six) on either side of the FA cation (Stranger *et al.*, 1978 ▸). Additionally, C2, in brown, appears displaced from the plane of the terminal iodide ions of the bi­octa­hedra, also resulting in a cubocta­hedral coordinated FAI_12_ environment.

The atomic positions, bi­octa­hedral bond lengths and angles of FA_3_Sb_2_I_9_ are summarized in Table 3 ▸. For FA_3_Sb_2_I_9_, the Sb—I1 bond length is 3.2323 (12) Å, while Sb—I2 is 2.8758 (13) Å. These align well with the Sb—I bond lengths of the structure that Szklarz *et al.*, (2020 ▸) collected on FA_3_Sb_2_I_9_ at 195 K, *i.e.* 3.213 and 2.881 Å. These values are also similar to those in the bi­octa­hedra of *A*_3_Sb_2_I_9_ (*A* = Cs or MA), with bond lengths of 3.198 and 2.870 Å for *A* = Cs, and 3.213 and 2.887 Å for *A* = MA (Chabot & Parthé, 1978 ▸; Ju *et al.*, 2018 ▸). These confirm crystallization of *A*_3_Sb_2_I_9_ in a Cs_3_Cr_2_Cl_9_-type crystal structure at room tem­per­a­ture. Bond angles in the range 83–94° for terminal I—Sb—I, and around 172.9° for bridging I—Sb—I are similarly com­parable to literature values. For MA_3_Sb_2_I_9_, the bond angles are in the range 84–91° for terminal I—Sb—I, and around 173.8° for bridging I—Sb—I. These angles diverge from 90 and 180°, respectively, indicating the distortion of the bi­octa­hedral units as a result of face-sharing in the *ab* plane.

**Figure 4:**
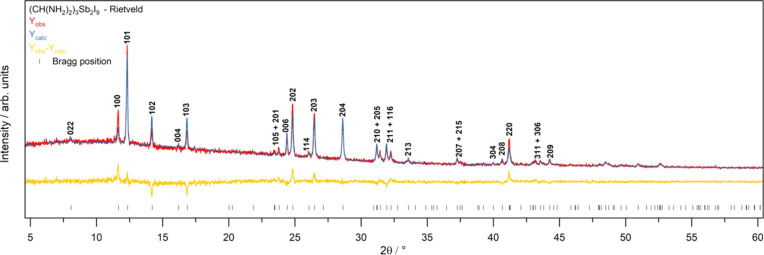
Rietveld refinement on the PXRD pattern of FA_3_Sb_2_I_9_ single crystals made by CDCG. Input unit-cell information was taken from the structure resolved from SCXRD data. Y_obs_ (red) is the collected diffraction pattern, Y_calc_ (blue) is the calculated pattern from *TOPAS-Academic* and Y_obs_ − Y_calc_ (yellow) is the residual plot. Reflections that are ≥5% of the highest intensity reflection are indexed.

The Rietveld refinement of PXRD data in Fig. 4 ▸ is based on the SCXRD structure and shows good agreement with the PXRD data collected, fortifying the triple-per­ov­skite structure at room tem­per­a­ture. A goodness-of-fit (GOF) of 1.11 and *R*_w_ = 9.217% were achieved.

X-ray photoelectron spectroscopy (XPS) was employed for the identification of chemical environments and elemental qu­anti­fication to verify the formation of *A*_3_*B*_2_*X*_9_ com­pounds; see Fig. S2 and Tables S1 and S2 in the supporting information. The elemental qu­anti­fication in Table S2 matches closely the expected *A*_3_*B*_2_*X*_9_ com­position. From the survey spectra [Fig. S2(*a*)], core lines from the expected elements for FA_3_Sb_2_*X*_9_ are observed, in addition to Si from contamination during sample plating. The supporting information also reports the absolute binding energy (BE) values of the core levels in Table S1, due to issues noted for the application of charge com­pensation (such as C 1*s*) for organic–inorganic com­pounds (Jia *et al.*, 2023 ▸). From the XPS spectra, the relative BE (ΔBE) between the N1*s* core line (from the FA cation, appearing around 400 eV) and the Sb 3*d*_5/2_ core line (appearing around 530 eV) is 130.2 eV for FA_3_Sb_2_Br_9_ and 129.6 eV for FA_3_Sb_2_I_9_. The values are within 1 eV of each other, indicating the presence of similar *A*- and *B*-site chemical environments, regardless of the halide. The reduction in the ΔBE(Sb 3*d*_5/2_—N1*s*) values from FA_3_Sb_2_Br_9_ to FA_3_Sb_2_I_9_ may be due to a shift of Sb3 *d*_5/2_ to lower BE and/or a shift of N 1*s* to higher BE. A shift of a core level to lower (higher) BEs is indicative of higher (lower) charge densities around the atom (Greczynski & Hultman, 2022 ▸). Given the lower electronegativity of iodine, it is reasonable to assume that the Sb in FA_3_Sb_2_I_9_ has greater charge density than Sb in FA_3_Sb_2_Br_9_, which then explains the change in ΔBE(Sb 3*d*_5/2_—N 1*s*).

## Conclusions and future perspectives

Single crystals of FA_3_Sb_2_*X*_9_ (*X* = Br^−^ and I^−^) were grown successfully by counter diffusion crystal growth in silica gel, and their room-tem­per­a­ture structures identified. FA_3_Sb_2_Br_9_ crystallizes in the Cs_3_Bi_2_Br_9_ structure type, while FA_3_Sb_2_I_9_ belongs to the Cs_3_Cr_2_Cl_9_ structure type. Both structures were com­pared to known com­pounds of group 15 triple-per­ov­skites. Further work to understand these crystal structures may include the study of tem­per­a­ture-dependent phase transitions, in particular to resolve the organic *A*-site positions and to gain further insights on the stability of these com­pounds.

## Related literature

The following references are cited in the supporting information for this article: Kalha *et al.* (2020 ▸); McCall *et al.* (2017 ▸); Scofield (1973 ▸); Scholz *et al.* (2018 ▸); Teterin *et al.* (2008 ▸); Wolstenholme (2008 ▸).

## Supplementary Material

Crystal structure: contains datablock(s) I, II, global. DOI: 10.1107/S2053229626000811/yd3067sup1.cif

Structure factors: contains datablock(s) I. DOI: 10.1107/S2053229626000811/yd3067Isup2.hkl

Structure factors: contains datablock(s) II. DOI: 10.1107/S2053229626000811/yd3067IIsup3.hkl

Treatment of the disordered formamidinium cation in single crystal refinement and additional experimental methods. DOI: 10.1107/S2053229626000811/yd3067sup4.pdf

Data for this article, including all processed data of the figures, are available at Zenodo in Origin format: https://doi.org/10.5281/zenodo.17578603

CCDC references: 2501007, 2500813

## Figures and Tables

**Table 1:** Experimental details Experiments were carried out at 295 K using an Agilent SuperNova Dual Source diffractometer with a HyPix-Arc 100 detector. Absorption was corrected for by multi-scan methods (*CrysAlis PRO*; Rigaku OD, 2022 ▸). Refinement was on 19 parameters. H-atom parameters were not defined.

	**FA_3_Sb_2_Br_9_**	**FA_3_Sb_2_I_9_**
Crystal data		
Chemical formula	(CH_5_N_2_)_3_[Sb_2_Br_9_]	(CH_5_N_2_)_3_[Sb_2_I_9_]
*M*_r_	998.72	1421.63
Crystal system, space group	Trigonal, *P* 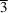 *m*1	Hexagonal, *P*6_3_/*mmc*
*a*, *b*, *c* (Å)	8.5161 (4), 8.5161 (4), 10.0380 (4)	8.7552 (4), 8.7552 (4), 21.8474 (12)
α, β, γ (°)	90, 90, 120	90, 90, 120
*V* (Å^3^)	630.46 (6)	1450.32 (15)
*Z*	1	2
Radiation type	Cu *K*α	Mo *K*α
μ (mm^−1^)	33.54	11.42
Crystal size (mm)	0.19 × 0.15 × 0.04	0.13 × 0.13 × 0.09

Data collection		
*T*_min_, *T*_max_	0.061, 1.000	0.263, 1.000
No. of measured, independent and observed [*I* > 2σ(*I*)] reflections	13069, 546, 391	36967, 896, 456
*R*_int_	0.089	0.104
(sin θ/λ)_max_ (Å^−1^)	0.631	0.728

Refinement		
*R*[*F*^2^ > 2σ(*F*^2^)], *wR*(*F*^2^), *S*	0.067, 0.241, 1.14	0.056, 0.242, 1.05
No. of reflections	546	896
No. of restraints	0	1
Δρ_max_, Δρ_min_ (e Å^−3^)	1.11, −1.03	1.24, −0.45

Computer programs: *CrysAlis PRO* (Rigaku OD, 2022 ▸), *SHELXT2014* (Sheldrick, 2015*a* ▸), *SHELXL2025* (Sheldrick, 2015*b* ▸), *OLEX2* (Dolomanov *et al.*, 2009 ▸), *VESTA* (Momma & Izumi, 2011 ▸) and *OLEX2* (Dolomanov *et al.*, 2009 ▸).

**Table 2:** Atomic position parameters, inter­atomic distances (Å) and bond angles (°) obtained from the structural solution of SCXRD data collected at 295 K of FA_3_Sb_2_Br_9_ *x*, *y* and *z* are the position parameters of different atoms, and ‘Occ’ is the occupancy of the atom at the determined position. W is the Wyckoff position notation and *U* is the anisotropic displacement parameter. C atoms are used in place of the FA ion, as explained in the text. Bond distances and angles were determined using the *VESTA* software suite (Version 3; Momma & Izumi, 2011 ▸).

Atom	*x*	*y*	*z*	Occ	W	*U*
Sb	1/3	2/3	0.31852 (12)	1	2*d*	0.1112 (8)
Br1	1/2	3/2	1/2	1	3*f*	0.1457 (11)
Br2	0.6278 (3)	0.81392 (16)	0.17401 (18)	1	6*i*	0.1709 (12)
C1	2/3	4/3	0.191 (4)	1	2*d*	0.21 (3)
C2	−3.00000	−1.00000	1/2	1	1*b*	0.22 (3)
						
Atoms		Inter­atomic distance		Atoms		Bond angle
Sb—Br1		3.0598 (7)		Br1—Sb—Br2		177.20 (6)
Sb—Br2		2.6119 (19)		Br1—Sb—Br1		88.18 (3)
Br1—Br2		4.016 (3)		Br2—Sb—Br2		92.14 (8)

**Table 3:** Atomic position parameters, inter­atomic distances (Å) and bond angles (°) obtained from the structural solution of SCXRD data collected at 295 K of FA_3_Sb_2_I_9_ See Table 2 ▸ for definitions.

Atom	*x*	*y*	*z*	Occ	W	*U*
Sb	2/3	1/3	0.34482 (6)	1	4*f*	0.0810 (6)
I1	0.50288 (8)	0.49712 (8)	1/2	1	6*h*	0.1312 (7)
I2	0.82610 (9)	0.65220 (18)	0.41593 (6)	1	12*k*	0.1312 (7)
C1	1/3	2/3	0.405 (4)	1	4*f*	0.21 (2)
C2	1.00000	1.00000	1/4	1	2*b*	0.20 (3)
						
Atoms		Inter­atomic distance		Atoms		Bond angle
Sb—I1		3.2323 (12)		I1—Sb—I2		172.94 (5)
Sb—I2		2.8758 (13)		I1—Sb—I1		83.41 (3)
I1—I2		4.3762 (15)		I2—Sb—I2		93.50 (5)

## Data Availability

Data for this article, including all processed data of the figures, are available at Zenodo in Origin format at https://doi.org/10.5281/zenodo.17578603.
