# Supplementary material for: Room-tem­per­a­ture crystal structures of [CH(NH2)2]3Sb2X9 (X = Br and I)
Source: Acta Crystallogr C Struct Chem. 2026 Feb 26;82(Pt 3):138–43. doi: 10.1107/S2053229626000811 (PMC12961747; doi:10.1107/S2053229626000811)
Supplement: Supplementary file 4 [file c-82-00138-sup4.pdf]

STRUCTURAL CHEMISTRY

Volume 82 (2026)

Supporting information for article:

Room-temperature crystal structures of $[CH(NH_2)_2]_3Sb_2X_9$ (X = Br and I)

Prajna Bhatt, Yuhan Liu, Anna Regoutz and Robert G. Palgrave

S1. Treatment of the disordered Formamidinium cation in single crystal refinement

The organic formamidinium (FA) cations are located within the cavities of the $[Sb_2X_9]^{3-}$ (X = Br, I) framework. Initial refinement cycles showed significant residual electron density in these regions; however, the density was featureless, preventing location of the discrete carbon and nitrogen atoms or the modelling of specific orientations. To account for this contribution more accurately, the solvent mask calculation in Olex2 was implemented (Dolomanov *et al.*, 2023). For $FA_3Sb_2Br_9$, the R-factor improved from 0.0668 to 0.0447, when a void of 244 $Å^3$ (38.7% of the total unit cell) that contains 77 electrons is identified. For $FA_3Sb_2I_9$, a void volume of 541 $Å^3$ per unit cell was identified (37.3% of the total unit cell), which contains 105 electrons. While this value is numerically lower than the theoretical count for six FA cations, it supports the existence of the disordered organic groups.

Additionally, the R-factors improved noticeably when high-angle reflections are ignored during refinement, from 0.0668 to 0.0544 for $FA_3Sb_2Br_9$ and from 0.0564 to 0.0473 for $FA_3Sb_2I_9$. However, it was decided to retain the full data range for the final reporting. The relatively large displacement parameters observed are likely to be a mathematical consequence of this resolution decay and the spatial averaging caused by the disordered cations.

Regarding the potential effects of secondary extinction, the SHELXL EXTI instruction was also tested for both the bromide and iodide structures to determine if it would improve the model fit. For $FA_3Sb_2Br_9$, the application of an extinction coefficient of 0.0022(10) resulted in a negligible decrease in the R-factor from 0.0668 to 0.0660. In the case of $FA_3Sb_2I_9$, the refinement of this parameter yielded a value of 0.0(4) and actually led to a slight increase in the R-factor from 0.0564 to 0.0578. Because the inclusion of an extinction correction did not provide a statistically significant improvement or a more physically reasonable model, it was omitted from the final refinements to maintain a more parsimonious parameter set.

Hence, for the final structure, a single carbon atom was utilized as a placeholder to represent the central density of the FA group. The highest residual peak is observed next to the carbon atom in both structures, indicating the residual density of FA cation, and confirming the FA cation's location.

S2. Additional Experimental Methods

X-ray photoelectron spectroscopy (XPS) was conducted on a Thermo Scientific^{TM} NEXSA spectrometer at HarwellXPS (Didcot, UK) the EPSRC National XPS Facility. The instrument employs a 72 W monochromatised Al Kα photon excitation source 1486.6 eV (1.47 keV), a hemispherical analyser and two–dimensional detector. The electron energy analyser consists of a double focusing 180° hemisphere with a mean radius 125 mm, operated in constant analyser energy (CAE) mode, and a 128 channel position sensitive detector Measurements were conducted with a 400 µm spot size, using a dual

beam flood gun (electron and Ar^{+} ion) with 100 mA current to provide charge compensation. The base pressure of the spectrometer was 9×10^{-8}mbar and the pressure during measurements was 5×10^{-7} mbar. Pass energies for surveys and core level spectra were 200 and 50 eV respectively. The experimental resolution of 560 meV was determined from Au foil, using the international standard method of resolution determination(Wolstenholme, 2008). For plotting, the BEs have been referenced to the halide core levels: Br 3*d* (68.3 eV) and I 3*d* (618.7 eV) and intensities normalised to the feature of maximum intensity in each spectrum, after the removal of a constant linear background.

The processing of the spectra was performed using the Thermo Scientific Avantage v.5.9925 software suite. The peak fitting functionality was used for elemental quantifications (using Scofield photoionsation cross sections) and determination of BE positions of peaks (Scofield, 1973 & Kalha, 2020). The in-built Smart background was applied which is a development of the Shirley background (Wolstenholme, 2008). For the spectral peaks, pseudo-Voigt functions based on the convolution of the Lorentzian and Gaussian contributions were used and in-built peak positions and areas ratios for doublet core levels were applied.

Raman spectra were collected on a Bruker SENTERRA II Raman microscope with the 633 nm laser excitation using the 50× objective magnifier and collected between 50 cm^{-1} and 500 cm^{-1} with a spectral resolution of 4 cm^{-1}. Scan iterations were maintained to 3 for each spectrum.

Measurements of diffuse reflectance spectroscopy (DRS) were done using a Shimadzu Scientific Instruments UV-2700i spectrophotometer which employs a double monochromator, with deuterium and tungsten-halogen lamps and was equipped with an integrating grating sphere module. DRS was collected from 185 to 900 nm. Samples were ground into a fine powder using a mortar and pestle and loaded onto pressed barium sulphate ($BaSO_4$) disks.

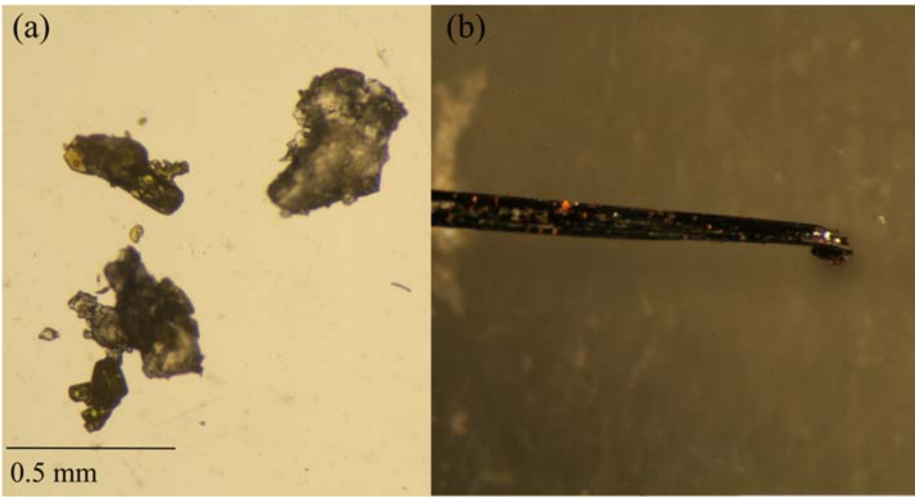

Figure S1 Optical microscope images of $FA_3Sb_2X_9$ (FA = $CH(NH_2)_2$; X = Br, I) crystals made by CDCG. Showing (a) $FA_3Sb_2Br_9$ and (b) $FA_3Sb_2I_9$. The scale bar is for both images.

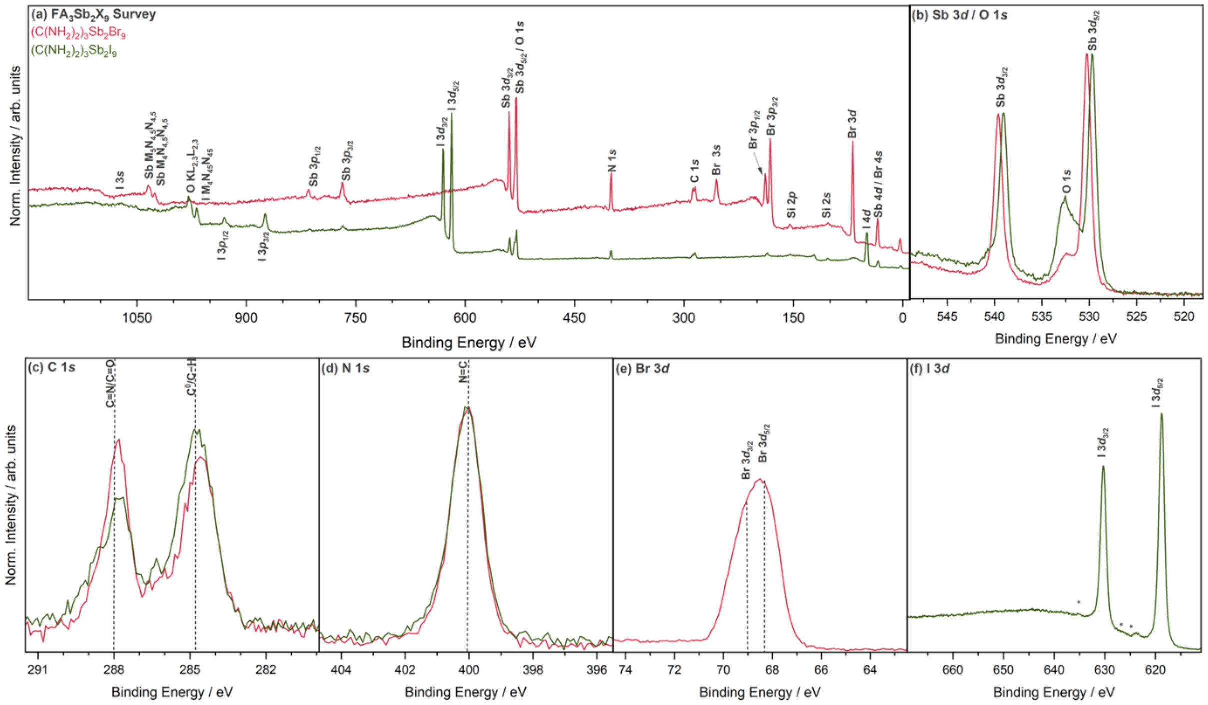

Figure S2 X-ray photoelectron spectra of $FA_3Sb_2X_9$ (FA = $CH(NH_2)_2$; X = Br, I) crystals made by CDCG. Spectra include (a) Survey, (b) Sb 3*d* / O 1*s*, (c) C 1*s*, (d) N 1*s*, (e) Br 3*d* and (d) I 3*d*.

Figure S2 depicts the survey and principle core level spectra of $FA_3Sb_2X_9$ (FA = $CH(NH_2)_2$; X = Br, I) crystals. The survey spectra in Figure S2 (a) confirm that the expected elements are present for the crystals. Additionally, oxygen (a-b) is present in all spectra originating from amorphous, adventitious species due to sample preparation and handling in ambient conditions (Greczynski & Hultman, 2022). Si is observed for $FA_3Sb_2Br_9$ due to the cross contamination during plating.

In photoelectron spectra for semiconductors, symmetrical peak shapes suggest singular chemical environments, seen for all high intensity features of the principle core levels (b-f). In (c), the lowest BE peak in C 1s is classified as C^0 / C—H, from the adventitious carbon and formamidinium ion (Teterin et al., 2008). The C=N species is located at higher BEs, as the bonded nitrogen has a higher electronegativity than carbon, which lowers the kinetic energy of an emitted photoelectron. Accordingly, the N 1s of alkylammonium N=C is observed in (d). Elemental quantification from the N 1s, Sb $3d_{3/2}$ and X $3d_{5/2}$ (X = Br, I) levels are summarised in Table S1. The expected ratio of N:Sb:X for these compounds is 6:2:9, which is seen, after accounting for any unwashed surface species on crystals.

Table S1 Absolute binding energy positions (in eV) of various core levels of $FA_3Sb_2X_9$ (FA = $CH(NH_2)_2$; X = Br, I) crystals made by CDCG. The error associated with values reported is ±0.2 eV.

Compound	N 1*s*	Sb $3d_{5/2}$	X $3d_{5/2}$	O 1*s*	C 1*s* (C^0/C—H)	C 1*s* (C=N/C—O)
$FA_3Sb_2Br_9$	399.8	530.0	68.2	532.4	284.3	287.6

$FA_3Sb_2I_9$	399.8	529.4	619.1	532.3	284.4	287.4

Table S2 Element quantification (in rel. at. %) based on peak fit analysis of the core levels of $FA_3Sb_2X_9$ (FA = $CH(NH_2)_2$; X = Br, I) crystals made by CDCG. The error associated with quantification is ±1 at. %. Quantification was conducted on the Avantage Software suite version 5.9925.

Compound	N 1*s*	Sb 3$d_{3/2}$	X 3$d_{5/2}$
$FA_3Sb_2Br_9$	31.4	10.1	52.4
$FA_3Sb_2I_9$	35.2	8.4	56.4

The elemental quantification in Table S2 matches closely the expected $A_3B_2X_9$ composition, where the N:Sb ratio for both compounds are approximately 3:1. Since each FA cation contains two N atoms, the elemental quantification form XPS supports the nominal ratio of N:Sb of 6 N and 2 Sb atoms per molecule of the triple-perovskite. N 1*s* has been used for quantification based on work done elsewhere ()

Raman spectra for $FA_3Sb_2X_9$ are identical to their methylammonium analogues, $MA_3Sb_2X_9$ that have been discussed in the main paper as isostructural compounds to crystals reported here (Scholz et al., 2018; McCall et al., 2017).

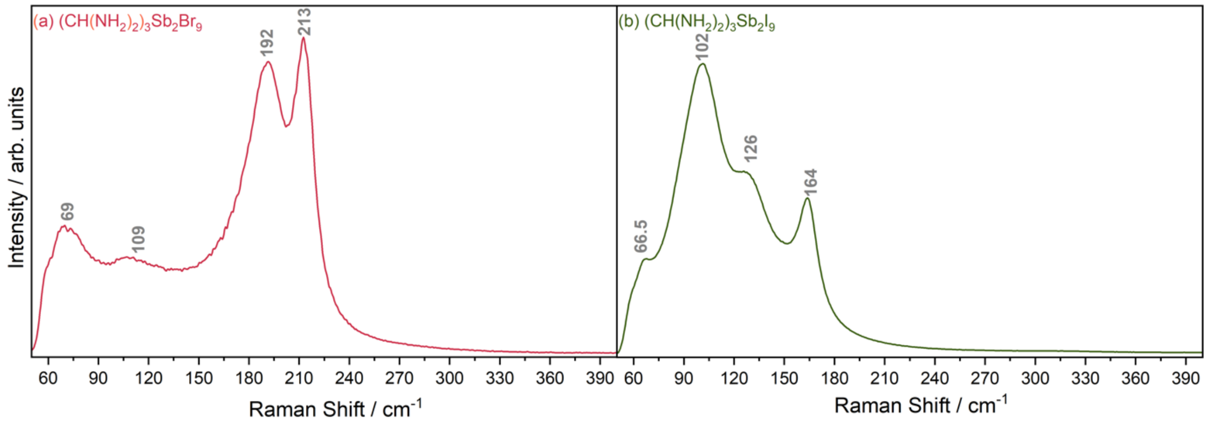

Figure S3 Raman spectra of crystals produced by CDCG including (a) $FA_3Sb_2Br_9$ and (b) $FA_3Sb_2I_9$. Spectra were collected using a 633 nm laser excitation source.

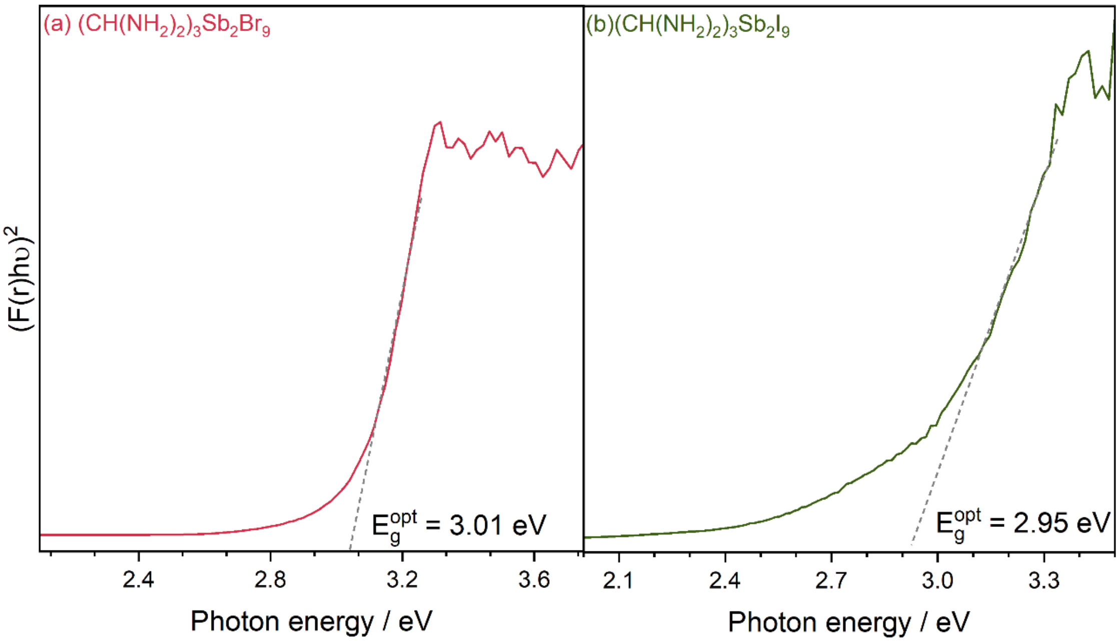

Figure S4 Tauc plot of crystals from DRS produced by CDCG including (a) $FA_3Sb_2Br_9$ and (b) $FA_3Sb_2I_9$.The E_g^{opt} or optical bandgaps are given in the plot.

References

Dolomanov, O. V., Bourhis, L. J., Gildea, R. J., Howard, J. A. & Puschmann, H. (2009) *J. Appl. Crystallogr*. **42.** 339–341.

Greczynski, G. & Hultman, L. (2022). *Vacuum.* **205.** 111463.

Kalha, C., Fernando, N. K. & Regoutz, A. (2020) *Digitisation of Scofield Photoionisation Cross Section Tabulated Data*, *figshare*, Dataset.

McCall, K. M., Stoumpos, C. C., Kostina, S. S., Kanatzidis, M. G. & Wessels, B. W. (2017) Chem. Mater. **29.** 4129–4145.

Scofield, J.H. (1973) *Technical Report UCRL-51326*, Lawrence Livermore Laboratory.

Scholz, M., Morgenroth, M., Oum, K. & Lenzer, T. (2018). *J. Phys. Chem. C.* **122.** 5854–5863.

Teterin, A. Y., Teterin, Y. A., Maslakov, K., Yarzhemskii, V., Sverchkov, S., Denker, B., Galagan, B., Iskhakova, L., Bulatov, L., Dvoirin, V. *et al.* (2008). *Dokl. Phys*. 2008, **53.** 566–570.

Wolstenholme, J. (2008). *Surf. Interface Anal.* **40.** 966–968.
